# Supplementary material for: Simulating molecular polaritons in the collective regime using few-molecule models
Source: Proc Natl Acad Sci U S A. 2023 Apr 6;120(15):e2219223120. doi: 10.1073/pnas.2219223120 (PMC10104552; doi:10.1073/pnas.2219223120)
Supplement: Supplementary file 1 — Appendix 01 (PDF) [file pnas.2219223120.sapp.pdf]

Supporting Information for

Simulating molecular polaritons in the collective regime using few-molecule models

Juan B. Pérez-Sánchez, Arghadip Koner, Nathaniel P. Stern and Joel Yuen-Zhou

Joel Yuen-Zhou.
E-mail: joelyuen@ucsd.edu

This PDF file includes:

Supporting text
Figs. S1 to S3

Supporting Information Text

Polariton Vibrational Relaxation.

Zeroth-Order Approximation:* N*-Independent Effects. Using Eqs. 17 and 29 in the main text, the model of relaxation in the zeroth-order approximation is given by the unperturbed and vibronic coupling Hamiltonians

$$\hat{\hat{H}}^{(0)} = \hat{\hat{H}}_0^{(0)} + \hat{\hat{H}}_I^{(0)},$$

$$\begin{aligned}\hat{\hat{H}}_0^{(0)} &= \omega|1,0\rangle\langle 1,0| + \left(\omega + \sum_k \omega_{\nu,k}\hat{b}_k^{\dagger}\hat{b}_k\right)|e\rangle\langle e| \\ &+ \sqrt{N}g\left(|e,0\rangle\langle 1,0| + |1,0\rangle\langle e,0|\right), \\ \hat{\hat{H}}_1^{(0)} &= \sum_k \omega_{\nu,k}\sqrt{s_k}\left(\hat{b}_k^{\dagger} + \hat{b}_k\right)|e\rangle\langle e|, \end{aligned} \quad [1]$$

where we have used the Fock basis for the vibrational bath (the second index “0" means all vibrational modes k are empty). The eigenstates of $\hat{\hat{H}}_0^{(0)}$ are trivial,

$$\begin{aligned}|\pm,0\rangle &= \frac{1}{\sqrt{2}}\left(|e,0\rangle \pm |1,0\rangle\right) \\ |D,m\rangle &= |e,m>0\rangle,\end{aligned} \quad [2]$$

where $m > 0$ denotes that at least one mode of the vibrational bath is not in the vacuum state. The eigenvalues are given by $\omega_{\pm,0} = \omega \pm g$ and $\omega_{D,m} = \omega + \sum_k \omega_{\nu,k}m_k$ respectively. Using Fermi's Golden Rule we can obtain the following relaxation rates:

$$\begin{aligned}\Gamma_{D\leftarrow+} &= 2\pi\sum_m |\langle D,m|\hat{\hat{H}}_1^{(0)}|+,0\rangle|^2\delta(\omega_{D,m} - \omega_{+,0}) \\ &= \frac{2\pi}{2}\sum_k \omega_{\nu,k}^2 s_k\delta(g - \omega_{\nu,k}),\end{aligned} \quad [3]$$

$$\Gamma_{-\leftarrow+} = 2\pi|\langle -,0|\hat{\hat{H}}^{(1)}|+,0\rangle|^2\delta(2g) = 0, \quad [4]$$

$$\Gamma_{-\leftarrow D} = 2\pi|\langle -,0|\hat{\hat{H}}^{(1)}|e,m\rangle|^2\delta(2g - \sum_k \omega_{\nu,k}m_k) = 0, \quad [5]$$

where the last two rates are equal to 0 because the resonance condition is not fulfilled. A schematic representation of the relaxation dynamics is shown in Fig. S1.

First-Order Correction:* $1/N$ *Effects. By analogy with the previous section, we describe the vibrations using the eigenbasis of the electronic ground-state vibrational Hamiltonians for the two molecules. Here we first rewrite Eq. 19 of the main text in terms of projection operators (see Eq. 6). The second label corresponds to the number of ground state molecules with phonons.

$$\begin{aligned}\hat{\hat{H}}^{(1)} &= \left(\mathbb{P}_1\hat{H}_{g,1}\mathbb{P}_1 + \mathbb{P}_2\hat{H}_{g,2}\mathbb{P}_2 + \omega_c\right)|1,0\rangle\langle 1,0| + \left(\hat{H}_{e,1} + \mathbb{P}_2\hat{H}_{g,2}\mathbb{P}_2\right)|e,0\rangle\langle e,0| \\ &+ \left(\mathbb{Q}_1\hat{H}_{g,1}\mathbb{Q}_1 + \mathbb{P}_2\hat{H}_{g,2}\mathbb{P}_2 + \omega_c\right)|1,1\rangle\langle 1,1| + \left(\mathbb{Q}_1\hat{H}_{g,1}\mathbb{Q}_1 + \hat{H}_{e,2}\right)|e,1\rangle\langle e,1| \\ &+ g\sqrt{N}\left(|e,0\rangle\langle 1,0| + |1,0\rangle\langle e,0|\right) + g\sqrt{N-1}\left(|e,1\rangle\langle 1,1| + |1,1\rangle\langle e,1|\right) + g\left(|e,0\rangle\langle 1,1| + |1,1\rangle\langle e,0|\right).\end{aligned} \quad [6]$$

Using Eqs. 6 and 29 in the main text, the relaxation model is described by the Hamiltonians in Eqs. 7.

The last two labels represent the vibrational states of molecules 1 and 2 in the Fock basis, $\hat{b}_{i,k}$ is the annihilation operator for the vibrational excitations of molecule i, and $m > 0$ means $m_k > 0$ for at least one mode k. The eigenstates of $\hat{\hat{H}}_0^{(1)}$ are given by

$$\begin{aligned}|\pm,0,0\rangle &= \frac{1}{\sqrt{2}}\left(|1,0,0,0\rangle \pm |e,0,0,0\rangle\right), \\ |\pm,m>0,0\rangle &= \frac{1}{\sqrt{2}}|1,1,m>0,0\rangle \\ &\pm \frac{1}{\sqrt{2}}\left(\sqrt{\frac{N-1}{N}}|e,1,m>0,0\rangle + \frac{1}{\sqrt{N}}|e,0,m>0,0\rangle\right), \\ |D,m>0,0\rangle &= \frac{1}{\sqrt{N}}|e,1,m>0,0\rangle + \sqrt{\frac{N-1}{N}}|e,0,m>0,0\rangle, \\ |D,m>0,n\rangle &= |e,1,m>0,n>0\rangle,\end{aligned}$$

$$
\begin{aligned}
\hat{\tilde{H}}^{(1)} &= \hat{\tilde{H}}^{(1)}_0 + \hat{\tilde{H}}^{(1)}_1, \\
\hat{\tilde{H}}^{(1)}_0 &= \omega|1,0,0,0\rangle\langle 1,0,0,0| + \sum_m \left(\omega + \sum_k \omega_{\nu,k} m_k\right)|e,0,m,0\rangle\langle e,0,m,0| + \sum_{m>0}\left(\omega + \sum_k \omega_{\nu,k} m_k\right)|1,1,m,0\rangle\langle 1,1,m,0| \\
&+ \sum_{m>0,n}\left(\omega + \sum_k \omega_{\nu,k} m_k + \sum_k \omega_{\nu,k} n_k\right)|e,1,m,n\rangle\langle e,1,m,n| + g\sqrt{N}\left(|1,0,0,0\rangle\langle e,0,0,0| + |e,0,0,0\rangle\langle 1,0,0,0|\right) \\
&+ g\sqrt{N-1}\sum_{m>0}\left(|1,1,m,0\rangle\langle e,1,m,0| + |e,1,m,0\rangle\langle 1,1,m,0|\right) + g\sum_{m>0}\left(|e,0,m,0\rangle\langle 1,1,m,0| + |1,1,m,0\rangle\langle e,0,m,0|\right), \\
\hat{\tilde{H}}^{(1)}_1 &= \sum_k \omega_{\nu,k}\sqrt{s_k}\left(\hat{b}^\dagger_{1,k} + \hat{b}_{1,k}\right)|e,0\rangle\langle e,0| + \sum_k \omega_{\nu,k}\sqrt{s_k}\left(\hat{b}^\dagger_{2,k} + \hat{b}_{2,k}\right)|e,1\rangle\langle e,1|.
\end{aligned} \quad [7]
$$

with eigenvalues $\omega_{\pm,0,0} = \omega \pm g\sqrt{N}$, $\omega_{\pm,m,0} = \omega + \sum_k \omega_{\nu,k} n_k \pm g\sqrt{N}$, and $\omega_{D,m,n} = \omega + \sum_k \omega_{\nu,k} m_k + \sum_k \omega_{\nu,k} n_k$ respectively. We can use these states to calculate the rate from the upper polariton to the lower polariton using Fermi's Golden Rule to get

$$
\begin{aligned}
\Gamma_{-\leftarrow+} &= 2\pi \sum_m |\langle -,0,0|\hat{\tilde{H}}^{(1)}_1|+,0,0\rangle|^2 \delta\left(\omega_{-,0,0} - \omega_{+,0,0}\right) \\
&= \frac{2\pi}{4N}\sum_k \omega^2_{\nu,k} s_k \delta(2\sqrt{N}g - \omega_{\nu,k}).
\end{aligned} \quad [8]
$$

Similarly, we can recalculate the decay rate from the upper polariton into the dark states,

$$
\begin{aligned}
\Gamma_{D\leftarrow+} &= 2\pi \sum_m |\langle D,m,0|\hat{\tilde{H}}^{(1)}_1|+,0,0\rangle|^2 \delta(\omega_{+,0,0} - \omega_{D,m,0}) \\
&= \left(\frac{N-1}{N}\right)\pi \sum_k \omega^2_{\nu,k} s_k \delta(\sqrt{N}g - \omega_{\nu,k}).
\end{aligned} \quad [9]
$$

where we have recovered the $\frac{N-1}{N}$ factor missing from Eq. 3 in the zero-order approximation. The final state in the previous calculation can be used as initial state to describe the subsequent relaxation from the dark-states into the lower polariton,

$$
\begin{aligned}
\Gamma_{-\leftarrow D} &= 2\pi \sum_{m'} |\langle -,m',0|\hat{\tilde{H}}^{(1)}_1|-,0,0\rangle|^2 \delta(\omega_{-,0,0} - \omega_{D,m=1,0}) \\
&= \left(\frac{N-1}{N^2}\right)\pi \sum_k \omega^2_{\nu,k} s_k \delta(\sqrt{N}g - \omega_{\nu,k}).
\end{aligned} \quad [10]
$$

The schematic representation of relaxation mechanisms is shown in Figs. S2 and S3.

Nonstatistical Excited-State Dynamics. The Hamiltonian for a system with N_A molecules of species A and N_B molecules of species B inside of the cavity can be written in the zeroth-order approximation as in Eq. 11.

$$
\tilde{H}^{(0)} = \left(\begin{array}{c|ccc|ccc}
\omega_c & g_A\sqrt{N_A}\langle\varphi_1^{(A)}|\phi_1^{(A)}\rangle & \cdots & g_A\sqrt{N_B}\langle\varphi_1^{(A}|\phi_{m_A}^{(A)}\rangle & g_A\sqrt{N_A}\langle\varphi_1^{(B)}|\phi_1^{(B)}\rangle & \cdots & g_B\sqrt{N_B}\langle\varphi_1^{(B)}|\phi_{m_B}^{(B)}\rangle \\
\hline
g_A\sqrt{N_A}\langle\varphi_1^{(A)}|\phi_1^{(A)}\rangle & \omega_{eg,1}^{(A)} & \cdots & 0 & 0 & \cdots & 0 \\
\vdots & 0 & \ddots & \vdots & \vdots & \ddots & \vdots \\
g_A\sqrt{N_A}\langle\varphi_1^{(A)}|\phi_{m_1}^{(A)}\rangle & \vdots & \ldots & \omega_{eg,m_A}^{(A)} & 0 & \ldots & 0 \\
\hline
g_B\sqrt{N_B}\langle\varphi_1^{(B)}|\phi_1^{(B)}\rangle & 0 & \cdots & 0 & \omega_{eg,1}^{(B)} & \cdots & 0 \\
\vdots & \vdots & \ddots & \vdots & 0 & \ddots & \vdots \\
g_B\sqrt{N_B}\langle\varphi_1^{(B)}|\phi_{m_B}^{(B)}\rangle & 0 & \cdots & 0 & \vdots & \ldots & \omega_{eg,m_B}^{(B)}.
\end{array}\right) \quad [11]
$$

The molecular PESs are given in mass-weighted coordinates ($\mu = 1$), and the parameters are:

Example 1.- $V_{g0,j}(q_j) = \frac{1}{2}\omega_g^2 q_j^2$ where $j \in \{A, B\}$ denote the molecular species, $V_{e0,A}(q_A) = \frac{1}{2}\omega_e^2(q_A - d_A)^2 + \omega_{eg,1}^{(A)}$, and $V_{e0,B}(q_B) = e^{-a(q_B - d_B)} + \omega_{eg,1}^{(B)}$. For our simulations, we have chosen $\omega_g = \omega_e = 0.22$ eV, $d_A = d_B = 0.66$ Å, $a = 0.90$ Å^{-1},

$\omega_{eg,1}^{(A)} = \omega_{eg,1}^{(B)} = 2.2$ eV, and $g_A\sqrt{N_A} = g_B\sqrt{N_B} = 0.22$ eV. The cavity frequency (ω_c) is resonant with the Franck-Condon transition of both A and B with $\omega_c = \omega_{eg,1}^{(A)} + \frac{1}{2}\omega_e^2 d_A^2$ (see Fig. 5,1a in the main manuscript).

Example 2.- $V_{g0,j}(q_j) = \frac{1}{2}\omega_g^2 q_j^2$ and $V_{e0,B}(q_B) = e^{-a(q_B-d_{B,1})} + ce^{-b(q_B-d_{B,2})^2} + \omega_{eg,1}^{(B)}$. The excited state potential of molecular species B is a dissociative potential with a bump to resemble the PES of species A only at the Franck-Condon point. Otherwise, the parameters are $\omega_g = \omega_e = 0.22$ eV, $d_A = 0$, $a = 1.54$ Å^{-1}, $d_{B,1} = 0.16$ Å, $b = 11.02$ Å^{-2}, $c = 0.51$ eV and $d_{B,2} = 0.50$ Å, and $\omega_{eg,1}^{(B)} = 1.88$ eV. (see Fig. 5,2a in the main manuscript)

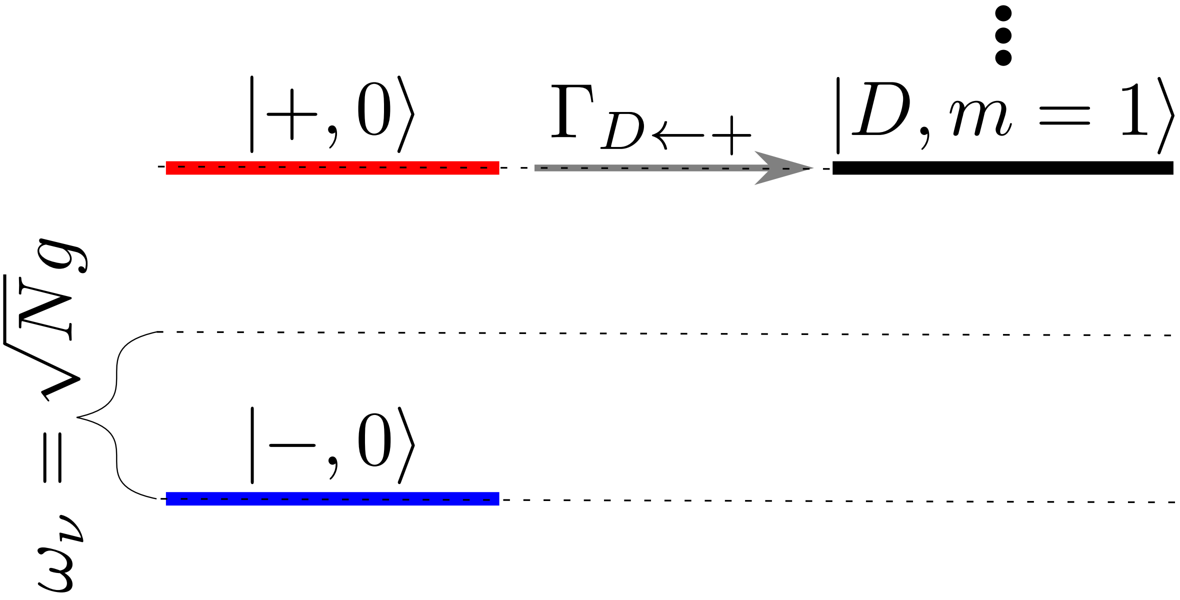

Fig. S1. Relaxation from the upper polariton to the dark states as described by the zeroth-order approximation (blue: lower polariton, red: upper polariton, black: dark states). States $|+,0\rangle$ and $|D,m=1\rangle$ are resonantly coupled through vibronic coupling. $m=1$ means there is 1 phonon in one of the bath modes of the excited effective molecule.

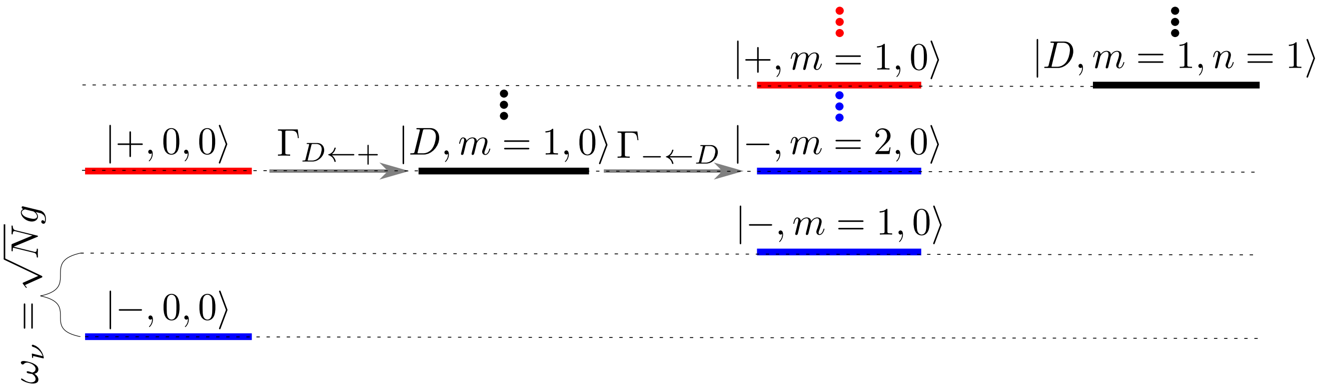

Fig. S2. Relaxation from the upper polariton to the lower polariton passing through the dark states, as described by inclusion of the first-order correction (blue: lower polariton, red: upper polariton, black: dark states). States $|+, 0, 0\rangle$, $|D, m=1, 0\rangle$, and $|-, m=2, 0\rangle$ are resonantly coupled through vibronic coupling. $m = 2$ means there are 2 phonons of frequency $\omega_\nu = \sqrt{N}g$ in the bath modes of the effective molecule 1. In the derivation, we have assumed the second phonon is emitted into a different bath mode than the first one.

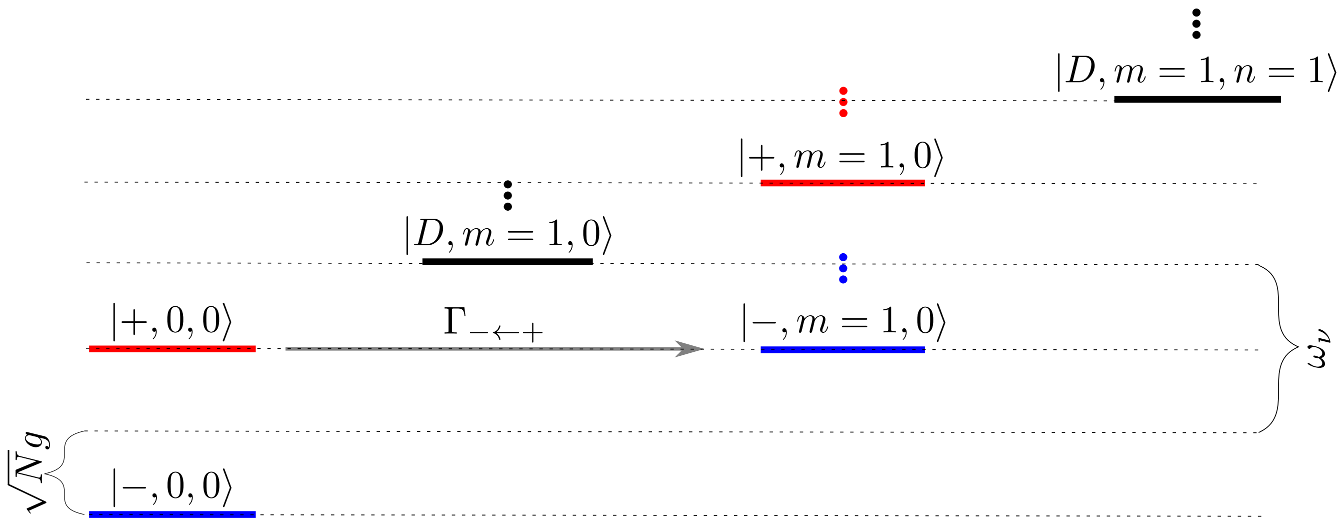

Fig. S3. Direct relaxation from the upper polariton to the lower polariton as described until the first-order correction (blue: lower polariton, red: upper polariton, black: dark states). States $|+, 0, 0\rangle$ and $|-, m = 1, 0\rangle$ are resonantly coupled through vibronic coupling to modes of frequency $\omega_\nu = 2\sqrt{N}g$.
